# Longitudinal Ellipsoid Zone Dynamics During Hydroxychloroquine Use

**DOI:** 10.3390/jpm15090416

**Published:** 2025-09-02

**Authors:** Karen Matar, Katherine E. Talcott, Obinna Ugwuegbu, Ming Hu, Sunil K. Srivastava, Jamie L. Reese, Justis P. Ehlers

**Affiliations:** 1 The Tony and Leona Campane Center for Excellence in Image-Guided Surgery and Advanced Imaging Research, Cole Eye Institute, Cleveland Clinic, Cleveland, OH 44195, USA; 2 Cole Eye Institute, Cleveland Clinic Foundation, Cleveland, OH 44195, USA; 3 Department of Quantitative Health Sciences, Lerner Research Institute, Cleveland Clinic Foundation, Cleveland, OH 44195, USA

**Keywords:** hydroxychloroquine retinopathy, ellipsoid zone integrity, optical coherence tomography, automated feature segmentation

## Abstract

**Background/Objectives**: Hydroxychloroquine (HCQ) retinopathy can be underrecognized early, as structural changes in OCT may precede symptoms and are often subtle. Early detection is crucial to prevent irreversible damage. This study evaluated longitudinal OCT changes preceding overt HCQ toxicity using ellipsoid zone (EZ) mapping. **Methods**: Patients on long-term HCQ underwent two macular cube scans at least one year apart using Cirrus HD-OCT. Scans were analyzed with an EZ-mapping platform and manually validated. Patients with baseline OCT signs of toxicity or co-existing macular disease were excluded based on masked expert review. **Results**: Three hundred and seventy-three eyes of 373 patients were included. The mean age was 57.0 ± 12.6 years, the mean HCQ dose was 379.4 ± 59.4 mg, the treatment duration was 5.6 ± 3.7 years, and the OCT interval was 3.1 ± 0.9 years. Outer retinal metrics remained stable across the cohort. The mean en face EZ attenuation increased from 3.3% to 3.9% (*p* = 0.24). Thirty-four eyes (9.1%) experienced an absolute increase of ≥4% (~1.5 mm^2^) in EZ attenuation. This increase was significantly associated with age at HCQ initiation (*p* < 0.001), age at the time of the first and second OCT (*p* < 0.001), and baseline visual acuity (*p* = 0.01), and demonstrated changes in other outer retinal metrics (*p* < 0.01). Only 3/34 eyes (8.9%) were diagnosed by the managing clinician with HCQ toxicity at the time of the second OCT. However, 26 of these eyes (76.5%) had signs of HCQ toxicity by expert review, suggesting the overall greater sensitivity of these quantitative outer retinal metrics for detecting toxicity compared with clinician review. **Conclusions**: Longitudinal OCT assessment revealed overall stability in outer retinal metrics in eyes on HCQ, but a subset showed increased EZ attenuation, which correlated with age at the time of HCQ initiation, baseline visual acuity, and expert OCT review. These changes may help identify at-risk eyes and eyes with early toxicity and warrant further validation as potential screening biomarkers.

## 1. Introduction

Hydroxychloroquine (HCQ) toxicity is associated with HCQ intake and can cause severe and permanent vision loss that can progress even after discontinuation of the medication [1,2,3]. Risk factors include an excessive daily dose (e.g., above the recommended dose based on actual body weight), cumulative dose, treatment duration over 10 years, advancing age, and concurrent macular disease [2,4,5,6,7,8,9,10]. Early detection of toxicity is key to stopping the drug and preventing severe vision loss. Unfortunately, no single test exists for identifying early toxicity. However, spectral-domain optical coherence tomography (SD-OCT) plays an important role in detecting photoreceptor and retinal pigment epithelium (RPE) involvement and can be used for both screening and monitoring purposes [2,7,11,12]. An important limitation of current OCT-based screening is the reliance on subjective image interpretation to identify subtle retinal changes, particularly given that OCT is often the frontline test performed by non-specialist eye care providers, such as comprehensive ophthalmologists and optometrists. Automated platforms for enhanced OCT assessment that enable detailed visualization of zonal changes of interest and provide objective quantitative metrics may improve the early identification of subclinical toxicity.

HCQ-related macular toxicity may be recognized in OCT through a range of well-documented structural changes, including parafoveal ellipsoid zone (EZ) disruption, thinning of the outer nuclear layer (ONL) and inner plexiform layer (IPL), peripapillary nerve fiber layer thinning, and the characteristic “flying saucer” sign [4,13,14,15]. Some studies have examined OCTs at later points in the disease following HCQ cessation with mixed results regarding stability in OCT findings, depending on the degree of disease severity [6,16,17,18]. Others have retrospectively evaluated OCT images from patients with known HCQ toxicity to identify early retinal changes that may precede the classic macular alterations. One study reported that thinning of the parafoveal ONL, disruption of the interdigitation zone, and decreased reflectivity of the parafoveal EZ may occur before overt parafoveal EZ loss [4]. Another investigation found that loss of a clearly continuous interdigitation zone could be an early OCT indicator of HCQ toxicity, even in patients with otherwise normal screening results, preceding more advanced parafoveal outer retinal damage and/or paracentral visual field defects [19]. These subtle changes can be quite difficult to subjectively identify, even with a baseline scan for comparison over time.

One way to better understand these early alterations would be to shift from subjective, qualitative assessment of outer retinal thinning to quantitative analysis of outer retinal metrics, supported by visual representation of EZ-RPE thickness maps [13,20,21,22]. A retinal segmentation and EZ-mapping platform has been previously described that provides in-depth outer retinal metrics [16,22,23,24]. Utilizing the EZ-mapping platform, alterations, such as partial EZ attenuation (the percentage of EZ-RPE thickness points ≤ 20 microns across the macular cube) and EZ-RPE central subfield thickness, have been associated with clinical outcomes and severity across various retinal disorders, including Stargardt disease, macular degeneration, ocriplasmin maculopathy, retinal vein occlusion, and diabetic macular edema [24,25,26,27,28,29]. Loss of EZ integrity in OCT is a hallmark feature of HCQ toxicity, but early alterations in the outer retina, including the EZ and interdigitation zone, can be subtle [13,16,20,21,22,23,24]. The EZ-RPE thickness metrics provide an important surrogate for photoreceptor outer segment (PROS) length/volume, a key anatomic marker of HCQ toxicity. Using this platform, outer retinal metrics have been quantified in both normal eyes and eyes with HCQ toxicity, including EZ-RPE thickness maps for visualization of annular thinning [16]. However, this platform has not been applied for longitudinal assessment of eyes in patients on HCQ on a large scale to explore potential subclinical outer retinal alterations and markers for toxicity risk [30].

Given the importance of identifying early and subtle OCT alterations in patients on HCQ, this study was initiated to explore longitudinal outer retinal metrics in the eyes of these patients. The purpose of this study was to evaluate longitudinal changes in OCT that may precede clinically recognized HCQ toxicity using an automated multi-layer segmentation and EZ-mapping platform.

## 2. Materials and Methods

This study was an institutional review board-approved retrospective case series of patients at the Cole Eye Institute, Cleveland Clinic, on HCQ who had longitudinal macular OCTs available for assessment. This study was conducted in accordance with the Declaration of Helsinki and all applicable Health Insurance Portability and Accountability Act regulations.

A medical record search was performed to identify patients seen for screening for HCQ toxicity, with OCT performed at two time points, with a minimum separation between testing of 1 year. All patients were on HCQ at the time of the first OCT until the second OCT. Patients with concurrent macular disease (e.g., age-related macular degeneration and diabetic macular edema) and those with evidence of HCQ toxicity at the baseline OCT were excluded. The process for determining baseline toxicity is described below. When patients had OCTs at more than two timepoints, the scans with the longest interval were selected. If both eyes were eligible, the right eye of each subject was used for analysis.

Baseline clinical characteristics were noted for patients, including age, gender, ethnicity, visual acuity, height, weight, co-existing kidney disease, and tamoxifen use. Follow-up clinical variables included age and visual acuity at the time of the second OCT time point. Specific clinical information regarding the HCQ usage was also noted, including daily dose and duration on medication. This information was used to calculate the cumulative dose, the dose based on actual body weight, and the dose based on ideal body weight.

**Figure 1:**
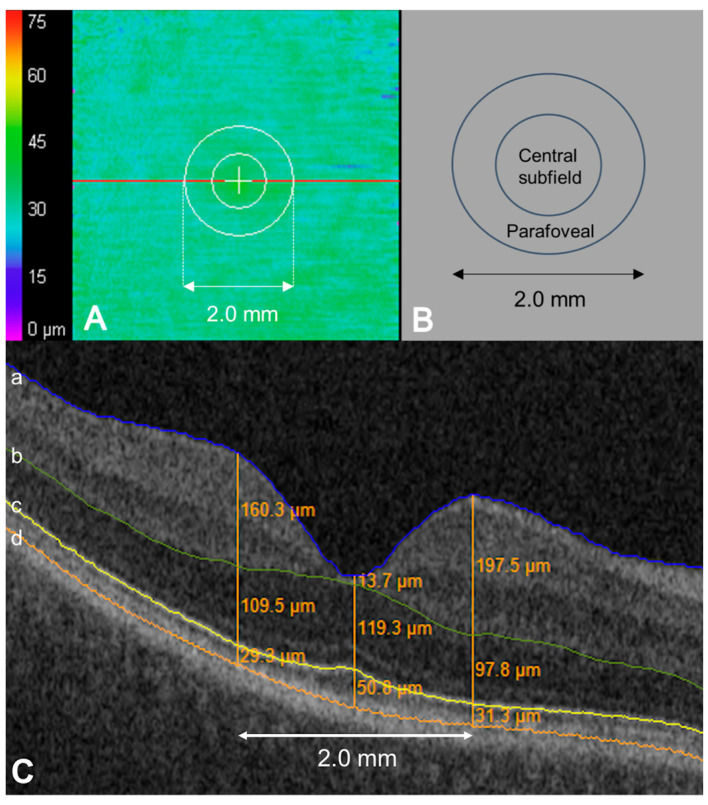
Ellipsoid zone (EZ) mapping and horizontal OCT B-scan image of hydroxychloroquine eye as an illustrative example of mapping and measurements. (**A**) EZ–retinal pigment epithelium (RPE) en face topographical map. The red line indicates the location of the horizontal B-scan. (**B**) Macular map sector with an inner circle representing the macular radius of 0.5 mm (corresponds to ‘central subfield’) and an outer circle representing the macular radius of 1.0 mm (corresponds to ‘parafoveal’). (**C**) Horizontal B-scan crossing the central fovea displaying semi-automatically segmented retinal layer boundaries. Each boundary was reviewed, and segmentation errors were manually corrected. The outer boundaries of the internal limiting membrane (a, blue), outer plexiform layer (b, green), EZ band (c, yellow), and RPE (d, orange). For retinal thickness measurement, three orange lines parallel to the vertical axis of the B-scan image are illustrated at the fovea, 1.0 mm nasal and temporal to the fovea. Orange figures indicate retinal thickness between the neighboring boundaries at each location.

All subjects underwent macular cube scans with a Cirrus HD-OCT (Zeiss, Oberkochen, Germany), with a raster scan pattern of 512 × 128 A-scans covering a 6 × 6 mm area centered on the fovea. SD-OCT scans were imported into the automated layer segmentation tool for outer retinal mapping and layer-based assessment, as previously described [23,24,26]. As shown in Figure 1, this software provides automated segmentation of the internal limiting membrane, the outer boundaries of the outer plexiform layer (ONL), the EZ band, and the RPE. Although segmentation was automated, all scans underwent full manual review and correction by trained image analysts to ensure accuracy. All image analysts underwent standardized training for OCT interpretation with a specific focus on outer retinal abnormalities. A senior image analyst provided quality assessment (QA) of every image frame following initial segmentation by the image analysts. Following the QA process, any additional questions regarding segmentation issues were referred to the principal investigator. Image analysts were masked to the clinical demographics and clinical features of the OCT scans.

En face EZ-RPE topographic thickness maps were generated to visualize the extent, severity, and location of EZ alterations, as previously described [16,23,24,26]. Data containing various outer retinal parameters were subsequently exported and evaluated. “Central subfield” and “parafoveal” were defined as a distance of 0.5 and 1.0 mm from the fovea, respectively. EZ-RPE represented the gap between the photoreceptor EZ band and the RPE mitochondria zone. The en face EZ-RPE topographic map provided calculations related to the percentage of total EZ attenuation/loss (e.g., the measurement value of EZ-RPE thickness of 0 µm) and partial EZ attenuation/degradation (EZ-RPE thickness ≤ 20 µm) across the macular cube, as previously described [16,23,24,26]. Specifically, the percentage measurement represents the percentage of thickness points in the en face map with a measurement (e.g., each A-scan) between the EZ-RPE of 20 or less microns for “partial attenuation” or 0 microns for “total attenuation.”

In addition to the overall mean changes across the cohort, a ≥4% increase in en face total EZ attenuation was used to define significant structural change. This threshold was derived from prior studies in which the mean en face partial EZ attenuation in mild HCQ toxicity differed from that of a normative database by approximately 4% [16]. Although not formally validated in this cohort, this threshold was selected beforehand to facilitate group-level comparisons and identify potentially at-risk individuals.

OCTs were also independently reviewed by up to two masked retina specialists at both OCT1 and OCT2. An initial screening of both scans for all eyes was performed by the first retina specialist. Eyes with suspected HCQ toxicity, concomitant macular disease, or a >4% longitudinal increase in partial EZ attenuation were pooled with control scans in a 3:1 fashion and independently reviewed by both retinal specialists in a masked fashion. Overall inter-reader agreement was strong (Cohen’s kappa coefficient = 0.8), and cases with disagreement were adjudicated by both retinal specialists in mutual review. Eyes with concomitant macular disease or toxicity at OCT1 were excluded to minimize confounding from pre-existing damage and to enable clearer assessment of longitudinal structural changes. Eyes with OCT features concerning HCQ toxicity at OCT2 were noted. Additionally, a chart review was also performed to determine if patients included in this study were considered to develop “clinical toxicity” by their provider over time, even following the OCT2 time point.

All statistical analyses were performed using R version 3.6.1. Longitudinal assessment of outer retinal parameters was compared from the first OCT to the second OCT using paired *t*-tests. Comparisons of significant findings with baseline characteristics were then performed using the Pearson correlation coefficient, chi-square test, and Fisher’s exact test. A *p*-value of <0.05 was considered statistically significant.

## 3. Results

Three hundred and seventy-three eyes from 373 patients on HCQ were included in this analysis. The baseline clinical characteristics are shown in Table 1. The mean age at the time of the first OCT was 57.0 ± 12.6 years. The majority of subjects were female (n = 322; 86%) and Caucasian (n = 260; 70%). Baseline visual acuity was excellent, with a mean of 20/25 (logMAR: 0.090 ± 0.17). The mean daily HCQ dose was 379.38.1 ± 59.40 mg, which corresponded to a mean actual body weight dose of 4.8 ± 1.5 mg/kg and a mean ideal body weight dose of 6.5 ± 1.5 mg/kg. Rheumatoid arthritis (n = 161; 43%) and lupus (n = 130; 35%) were the most common clinical indications for HCQ use, and risk factors for HCQ toxicity, including concurrent tamoxifen use (n = 7; 2%) and kidney disease (n = 24; 6%), were relatively rare. Subjects had been on HCQ, on average, for 5.6 ± 3.7 years at the time of the first OCT, and the mean time between the first and second OCTs was 3.1 ± 0.9 years.

**Figure 2:**
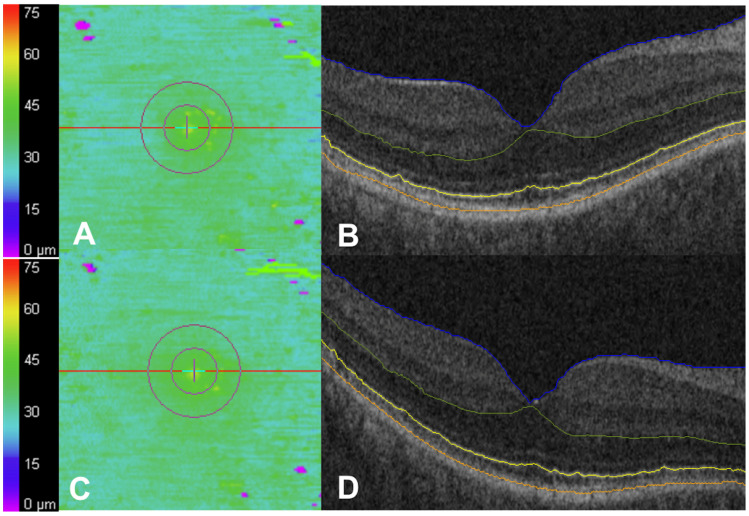
Case example of longitudinal stability while on hydroxychloroquine (HCQ). A 47-year-old woman on HCQ, where daily dosing based on actual body weight was 5.56 mg/kg/day, for 12 years at the time of the first OCT (**A**,**B**) and 14 years at the time of the second OCT (**C**,**D**). There were no significant changes in ellipsoid zone (EZ)–retinal pigment epithelium (RPE) mapping (**A**,**C**) or horizontal B-scan images (**B**,**D**). The outer boundaries of the internal limiting membrane (blue), outer plexiform layer (green), EZ band (yellow), and RPE (orange) (**B**,**D**).

The majority of subjects on HCQ demonstrated stability in outer retinal parameters, with a representative patient shown in Figure 2. Overall, there was no significant longitudinal change in the examined outer retinal metrics. There was a non-significant increase in partial EZ attenuation from the first OCT (3.3 ± 12.2%) to the second OCT (3.9 ± 12.0%; *p* = 0.24). There was no significant longitudinal change in the remainder of the outer retinal metrics, including EZ total attenuation, central subfield EZ-RPE thickness, central subfield EZ-RPE point nasal and temporal thickness, central subfield EZ-RPE volume, parafoveal EZ-RPE volume, parafoveal EZ-RPE point nasal and temporal thickness, parafoveal EZ-RPE thickness, and EZ-RPE volume (Table 2; all *p* > 0.05).

**Figure 3:**
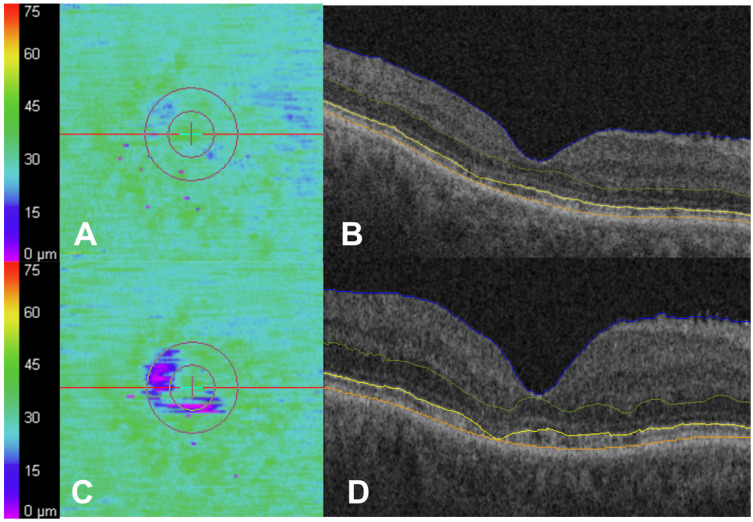
Case example of progressive en face ellipsoid zone (EZ) attenuation while on hydroxychloroquine (HCQ). A 70-year-old woman on HCQ. The daily dosing based on actual body weight was 5.22 mg/kg/day for 1 year at the time of the first OCT (**A**,**B**) and 6 years at the time of the second OCT (**C**,**D**). There were subtle areas of partial parafoveal ellipsoid zone (EZ)–retinal pigment epithelium (RPE) attenuation in the EZ-RPE mapping (**A**) and in the first OCT (**B**). These areas increased in size and became more confluent with progression to areas of total EZ-RPE attenuation in the EZ-RPE mapping (**C**) and in the second OCT (**D**). Subtle EZ attenuation may have been present in OCT at the first OCT (**B**) but became more apparent by the second OCT (**D**). The outer boundaries of the internal limiting membrane (blue), outer plexiform layer (green), EZ band (yellow), and RPE (orange) (**B**,**D**).

However, thirty-four eyes (9.1%) demonstrated a mean increase in partial EZ attenuation of more than 4% between the two OCT timepoints. This 4% threshold, previously associated with mild HCQ toxicity [16], is illustrated in a representative case shown in Figure 3. Among this group of 34 eyes, there was a mean increase in partial EZ attenuation of 17.6 ± 16.8% (*p* < 0.001) compared with the remaining eyes, which demonstrated no significant change in partial EZ attenuation (*p* = 0.37). Subjects with a ≥4% increase in partial EZ attenuation were older at the time of HCQ initiation (mean: 61.6 versus 50.5 years), the first OCT (mean: 68.0 versus 55.9 years), and the second OCT (71.0 versus 59.0 years) (all *p* < 0.001). It was also associated with visual acuity at the first OCT (mean logMAR VA of 0.20 or Snellen of 20/32 versus 0.08 or Snellen of 20/25; *p* = 0.01) and at the second OCT (mean logMAR VA of 0.20 versus 0.09; *p* = 0.02). It was not associated with ethnicity, gender, duration on HCQ, daily HCQ dose, actual or ideal body weight HCQ dose, cumulative HCQ dose, or time between OCTs (all *p* > 0.05). These results are summarized in Table 3. Additionally, patients who experienced a 4% or more increase in partial EZ attenuation had associated changes in other outer retinal metrics. There were significant differences in at least en face EZ total attenuation (Table 4; *p* < 0.001), EZ-RPE volume (*p* = 0.002), and parafoveal EZ-RPE thickness (*p* = 0.006) and volume (*p* = 0.004).

At the time of the second OCT or following, six eyes (1.6%) included in the series were clinically recognized by their treating provider as having HCQ toxicity. The last follow-up visit occurred at a mean of 3.6 years after the second OCT (range: 0 to 6.0 years). Three of these eyes were diagnosed at the time of the second OCT, and the other three at a time point following. OCT review alone was used to diagnose three of the eyes. The other three relied additionally upon Humphrey visual field (HVF) and multifocal electroretinogram. All the eyes clinically diagnosed with HCQ toxicity at the second OCT, and one of the eyes diagnosed later, had a 4% or more increase in EZ attenuation between the first and second OCTs. These eyes were also determined to have HCQ toxicity at the second OCT by the expert readers.

Among the subset of 34 eyes with a 4% or more increase in EZ attenuation, only 3 of these eyes (8.9%) were clinically recognized by their treating provider as having HCQ toxicity by the time of the second OCT. An additional eye (2.9%) was diagnosed with HCQ toxicity at a time point following the second OCT. Conversely, the expert readers determined that 26 of these eyes (76.5%) had HCQ toxicity based on review of the second OCT.

In the overall cohort of 373 eyes, the expert readers noted progression from the first OCT to the second OCT consistent with HCQ toxicity in 27 eyes (7.2%). A total of 3 of these 27 eyes (11.1%) were clinically recognized to have HCQ toxicity at the second OCT, and an additional eye (3.7%) following the second OCT. All eyes but one (96.3%) were noted to have at least 4% longitudinal progression of EZ attenuation.

## 4. Discussion

In this paper, automated quantification of outer retinal metrics was evaluated longitudinally in a large cohort of subjects on HCQ. These EZ-RPE thickness metrics provide an important surrogate for photoreceptor outer segment length/volume, a critical marker of HCQ toxicity. This assessment demonstrated overall stability in outer retinal metrics over a mean interval of approximately 3 years. However, a subset of patients showed progression. Specifically, 9.1% of eyes demonstrated a more than 4% absolute increase in partial EZ attenuation, a value previously associated with early HCQ toxicity [16]. This increase was associated with increased age at HCQ initiation and OCT, as well as worse baseline visual acuity. These eyes also had associated changes in other outer retinal metrics, including total EZ attenuation, EZ-RPE volume, and parafoveal EZ-RPE thickness and volume. Only three of these eyes were clinically diagnosed with HCQ toxicity at the time by their eye care providers, yet nearly 80% were determined to have signs of toxicity by expert retina specialist masked review. While progressive longitudinal OCT changes have been previously described in patients with known toxicity, the evaluation of such changes in a broader HCQ-treated cohort without baseline toxicity is unique [4,16,17,20,30].

This study expands on earlier work demonstrating the utility of using an automated EZ-mapping platform in detecting outer retinal changes in OCT in HCQ patients [16]. That platform was previously applied to patients with clinically evident HCQ toxicity and showed significant reductions in outer retinal parameters compared with controls, as well as a progressive decline in some metrics. In contrast, this study found overall stability, likely because patients with clinically recognized HCQ toxicity at baseline were excluded. Additionally, all OCTs underwent expert review to exclude eyes with co-existing macular disease or suspected toxicity. In some ways, this allowed for a relatively “clean” population of HCQ patients, which differs from routine practice, where subtle toxicity may be missed or difficult to ascertain due to concomitant OCT changes. Nevertheless, a subset of patients showed progressive longitudinal changes in OCT [16]. In particular, en face EZ attenuation maps showed patterns consistent with mild HCQ toxicity in previously described reports, including parafoveal thinning that became more pronounced over time [16].

Given the irreversible nature of HCQ retinopathy, early detection remains a major clinical priority. For instance, Lally et al. examined longitudinal OCTs of 30 eyes with HCQ retinopathy and found that parafoveal ONL thinning, disruption of the parafoveal interdigitation zone, and reduced reflectivity of the parafoveal EZ preceded parafoveal EZ disruption [4]. While this study focused on quantitative metrics, changes in partial EZ attenuation may reflect these earlier alterations, as loss of the interdigitation zone would likely lead to a reduction in EZ-RPE distance. Additionally, eyes with a significant increase in partial EZ attenuation in this study also showed changes in parafoveal EZ-RPE thickness and volume. Of note, they also found that the nasal inner subfield showed more thinning than temporally, similar to our trend toward a decrease in parafoveal EZ-RPE point nasal but not temporal thickness. Additionally, Garrity et al. reported on 10 patients with HCQ retinopathy with OCT changes but reassuring visual field tests, finding early OCT alterations of the attenuation of the parafoveal ellipsoid zone and loss of a continuous interdigitation zone [19]. Similar to Lally et al., these studies described subtle qualitative changes in OCT that can be easily missed and were identified retrospectively. These subtle changes may be more obvious to clinicians using automated segmentation with visualization of thickness maps, change analysis over time, and quantitative metrics.

Prior studies have shown that patients on HCQ exhibit stable OCT findings until they develop relatively rare retinopathy, rather than inevitably and gradually collecting toxic changes [20]. This current report echoes this, with most eyes demonstrating structural stability in the absence of baseline toxicity. Broader progression may have been observed if patients with undiagnosed toxicity or coexisting retinal disease were included, but these were excluded to reduce confounding and ensure a cleaner assessment of HCQ-associated changes. Still, a meaningful subset of eyes (9.1%) demonstrated significant progression, defined as a ≥4% absolute increase in partial EZ attenuation, a threshold previously associated with early HCQ retinopathy [16].

This EZ attenuation metric may represent a more sensitive marker for identifying early toxicity compared with traditional point thickness measures, which often remain unchanged until more advanced damage occurs. Importantly, all clinically diagnosed cases in this study exceeded the 4% threshold, and expert reviewers identified signs of toxicity in 76.5% of these cases, reinforcing the utility of this metric for early detection. The relatively large standard deviation observed in EZ attenuation change further highlights the heterogeneity of structural progression, likely influenced by variability in cumulative HCQ exposure, individual susceptibility, or baseline retinal status. These findings underscore the possibility that HCQ retinopathy may be underdiagnosed, which is not surprising given that signs of toxicity in OCT are subtle, and treating eye providers, including retinal specialists, general ophthalmologists, and optometrists, have varying levels of experience reading macular OCTs.

As such, there is a growing need for improved screening tools and predictive models to identify at-risk individuals earlier in the disease course. Advances in automated OCT analysis offer a promising avenue by enabling sensitive detection of structural change and quantification of EZ integrity [31,32,33]. Patients with increased EZ attenuation may benefit from more intensive surveillance or additional testing. However, in the absence of corresponding functional assessments, the clinical significance of these structural changes remains uncertain. Future prospective studies incorporating modalities such as visual fields (VFs), fundus autofluorescence (AF), and multifocal electroretinograms (mfERGs) are needed to validate these metrics as reliable biomarkers of HCQ toxicity.

There are several limitations to this study, largely resulting from its retrospective design. OCT orientation was not prospectively optimized, and significant tilt may result in alterations to EZ reflectivity and segmentation accuracy. Although the EZ-mapping platform provided automated segmentation, manual correction was required for all scans, limiting the current applicability of this approach in fully automated clinical screening settings. Further validation and development of scalable, automated methods are needed to enable broader clinical adoption. While inter-reader agreement was high (Cohen’s kappa = 0.8), intergrader reliability stratified by toxicity status was not assessed, limiting evaluation of reproducibility in a screening context. Importantly, functional tests such as VFs, AF, and mfERGs were not consistently available and were, therefore, not included. This limited our ability to correlate structural findings with visual function, constraining interpretation of EZ attenuation as a clinically validated biomarker. Additionally, the ≥4% threshold for defining significant change in partial EZ attenuation was derived from prior studies but has needs ongoing validation. Its diagnostic relevance remains uncertain, and future studies are needed to refine cut-offs and determine their relationship with functional outcomes. Only one eye per subject was included to avoid inter-eye correlation, but this may introduce selection bias and limits generalizability. Furthermore, only univariable analyses were performed; larger studies with more robust statistical power will be necessary to support multivariable regression and evaluate potential confounding factors. Due to the retrospective nature of this study, baseline OCTs from the time of HCQ initiation were available for all subjects. This is of particular interest given the slightly higher baseline partial EZ attenuation measurement compared with normal, which likely reflects underlying toxicity in select subjects [23]. Although eyes with overt HCQ toxicity were excluded based on masked expert OCT review, subclinical changes may have gone unrecognized at baseline, potentially biasing results toward apparent structural stability. This study also lacked a control group of HCQ-naïve or healthy subjects, which limits the ability to establish normative EZ metrics and assess the specificity of changes observed. Furthermore, the cohort was predominantly composed of white females, consistent with the epidemiology of autoimmune disease, but this demographic skew may affect generalizability. Finally, follow-up intervals varied across patients, and only two timepoints were analyzed per subject. Extended follow-up or inclusion of additional OCTs per patient may have provided further insight, but was limited by clinical availability. One additional important consideration is the multiple ways to consider EZ integrity, including preservation of relative reflectivity brightness. In this assessment, EZ-RPE thickness alterations were utilized as measures of EZ integrity. In our clinical experience, as the EZ-RPE compartment collapses, EZ brightness is often reduced as well, but future assessments of EZ reflectivity and correlation of EZ integrity with EZ-RPE thickness would be worthwhile.

Overall, this study offers a quantitative longitudinal assessment of EZ metrics in a large cohort of HCQ-treated patients. While most eyes without baseline toxicity demonstrated structural stability, a subset showed progressive en face EZ attenuation. These findings highlight the potential utility of this platform as a screening tool to detect subtle, early outer retinal changes that may precede overt toxicity. Finally, it also suggests that HCQ retinopathy may be underdiagnosed and a need for additional screening tests and metrics. Further studies are needed to further validate these longitudinal metrics and their impact on identification of HCQ retinopathy.

## Figures and Tables

**Table 1:** Baseline clinical characteristics of eyes on hydroxychloroquine who had spectral-domain OCT at two time points and were analyzed using a semi-automated ellipsoid-zone-mapping platform.

	Baseline Characteristics (n = 373 Eyes)
Age at first OCT, years	
Mean ± SD (median, range)	57.0 ± 12.6 (58, 14–89)
Gender	
N (percentage)	
Female	322 (86)
Male	51 (14)
Ethnicity	
N (percentage)	
White	260 (70)
Black	89 (24)
Hispanic	4 (1)
Asian	9 (2)
Other	11 (3)
Age HCQ initiated, years	
Mean ± SD	51.5 ± 12.5
Daily HCQ dose, mg	
Mean ± SD	379.38 ± 59.40
HCQ actual body weight dose, mg/kg	
Mean ± SD	4.8 ± 1.5
HCQ ideal body weight dose, mg/kg	
Mean ± SD	6.5 ± 1.5
Time between OCTs, years	
Mean ± SD	3.1 ± 0.9
Cumulative dose at first OCT, kg	
Mean ± SD	0.8 ± 0.5
Duration on HCQ, years	
Mean ± SD	
First OCT	5.6 ± 3.7
Second OCT	8.6 ± 3.8
LogMAR visual acuity	
Mean ± SD (median, range) (Snellen equivalent)	
First OCT	0.090 ± 0.17 (0, −0.12–1.3) (Snellen 20/25)
Second OCT	0.103 ± 0.18 (0, −0.12–2.2) (Snellen 20/25)
Longitudinal change	0.013 ± 0.22 (0, −1–1.90)
Tamoxifen use	
N (percentage)	
Yes	7 (2)
No	366 (98)
Kidney disease	
N (percentage)	
Yes	24 (6)
No	349 (4)
Rheumatoid arthritis	
N (percentage)	
Yes	161 (43)
No	212 (57)
Lupus	
N (percentage)	
Yes	130 (35)
No	243 (65)

SD = standard deviation, HCQ = hydroxychloroquine, OCT = optical coherence tomography, mg = milligram, and kg = kilogram.

**Table 2:** Longitudinal changes in outer retinal parameters over two OCTs in eyes on hydroxychloroquine.

	OCT 1 (Mean ± SD)	OCT 2 (Mean ± SD)	Difference (Mean ± SD)	*p*-Value (Paired *t*-Test)
Partial EZ attenuation (EZ -RPE thickness ≤ 20 µm; %)	3.3 ± 12.2	3.9 ± 12.0	0.6 ± 9.4	0.24
En face percentage of EZ total attenuation (EZ-RPE thickness = 0 µm; %)	2.1 ± 10.5	2.3 ± 9.0	0.2 ± 8.0	0.60
Central subfield EZ-RPE thickness (µm)	40.4 ± 4.6	40.3 ± 4.6	−0.12 ± 3.4	0.49
Central subfield EZ-RPE volume (mm^3^)	0.032 ± 0.004	0.032 ± 0.004	0.000 ± 0.003	0.40
Central subfield EZ-RPE point nasal thickness (0.5 mm from fovea; µm)	39.1 ± 5.3	38.6 ± 6.0	−0.48 ± 5.6	0.10
Central subfield EZ-RPE point temporal thickness (0.5 mm from fovea; µm)	37.9 ± 5.7	37.7 ± 5.7	−0.2 ± 6.0	0.49
Parafoveal EZ-RPE thickness (µm)	36.8 ± 3.8	36.6 ± 3.9	−0.2 ± 3.0	0.35
Parafoveal EZ-RPE volume (mm^3^)	0.12 ± 0.01	0.11 ± 0.01	0.00 ± 0.01	0.39
Parafoveal EZ-RPE point nasal thickness (1 mm from fovea; µm)	35.6 ± 5.1	35.1 ± 5.6	−0.5 ± 5.9	0.08
Parafoveal EZ-RPE point temporal thickness (1 mm from fovea; µm)	35.1 ± 5.9	35.1 ± 5.6	0.0 ± 6.1	0.96
EZ-RPE volume (mm^3^)	1.2 ± 0.2	1.2 ± 0.2	0.0 ± 0.1	0.53

OCT = optical coherence tomography, OCT 1 = first OCT, OCT 2 = second OCT, SD = standard deviation, EZ = ellipsoid zone, µm = micrometer, % = percent, RPE = retinal pigment epithelium, and mm3 = cubic micrometer. Central subfield is equivalent to a distance of 0.5 mm from the fovea (inner circle). Parafoveal is equivalent to a distance of 1.0 mm from the fovea (outer circle). Difference for en face percentage of attenuation is stated as an absolute change in percentage.

**Table 3:** Correlated variables among patients with a longitudinal increase in en face EZ attenuation.

	Patients with Increase in partial EZ Attenuation of 4% or greater (n = 34)	Patients with < 4% Increase in partial EZ Attenuation (n = 339)	*p*-Value (Paired *t*-Test)
Age HCQ initiated, years Mean ± SD	61.6 ± 13.0	50.5 ± 12.1	**<0.001**
Age at first OCT, years Mean ± SD	68.0 ± 12.8	55.9 ± 12.1	**<0.001**
Age at second OCT, years Mean ± SD	71.0 ± 13.3	59.0 ± 12.3	**<0.001**
Cumulative HCQ dose at first OCT, kg Mean ± SD	0.86 ± 0.58	1.28 ± 0.59	0.24
Cumulative HCQ dose at second OCT, kg Mean ± SD	1.31 ± 0.58	1.14 ± 0.74	0.12
Actual body weight HCQ dose, mg/kg Mean ± SD	5.11 ± 1.19	4.78 ± 1.56	0.15
Ideal body weight HCQ dose, mg/kg Mean ± SD	6.97 ± 1.38	6.50 ± 1.56	0.07
LogMAR visual acuity, first OCT Mean ± SD	0.20 ± 0.24	0.08 ± 0.16	**0.01**
LogMAR visual acuity, second OCT Mean ± SD	0.20 ± 0.25	0.09 ± 0.17	**0.02**

OCT = optical coherence tomography, EZ = ellipsoid zone, SD = standard deviation, kg = kilogram, mg = milligram, and HCQ = hydroxychloroquine.

**Table 4:** Correlated longitudinal change in outer retinal parameters among patients with a longitudinal increase in en face EZ attenuation.

	Patients with Increase in partial EZ Attenuation > 4% (n = 34; Mean ± SD)	Patients with <4% Increase in partial EZ Attenuation (n = 339; Mean ± SD)	*p*-Value (Paired *t*-Test)
Partial EZ attenuation (Longitudinal change; EZ-RPE thickness ≤ 20 µm; %)	17.6 ± 168	−1.1 ± 6.2	**<0.001**
Total EZ attenuation (Longitudinal change; EZ-RPE thickness = 0 µm; total loss; %)	11.8 ± 14.7	−0.9 ± 5.9	**<0.001**
Central subfield EZ-RPE thickness (Longitudinal change; µm)	−0.87 ± 3.24	−0.05 ± 3.39	0.17
Central subfield EZ-RPE volume (Longitudinal change; mm^3^)	−0.001 ± 0.003	0.000 ± 0.003	0.09
Central subfield EZ-RPE point nasal thickness (Longitudinal change; 0.5 mm from fovea; µm)	−2.9 ± 7.5	−0.2 ± 5.4	0.05
Central subfield EZ-RPE point temporal thickness (Longitudinal change; 0.5 mm from fovea; µm)	−0.8 ± 8.9	−0.2 ± 5.7	0.68
Parafoveal EZ-RPE thickness (Longitudinal change; µm)	−1.58 ± 3.05	0.00 ± 2.95	**0.006**
Parafoveal EZ-RPE volume (Longitudinal change; mm^3^)	−0.005 ± 0.010	0.000 ± 0.009	**0.004**
Parafoveal EZ-RPE point nasal thickness (Longitudinal change; 1 mm from fovea; µm)	−2.9 ± 8.8	−0.3 ± 5.5	0.10
Parafoveal EZ-RPE point temporal thickness (Longitudinal change; 1 mm from fovea; µm)	−0.81 ± 8.07	0.10 ± 5.91	0.53
EZ-RPE volume (Longitudinal change; mm^3^)	−0.05 ± 0.09	0.01 ± 0.08	**0.002**

OCT = optical coherence tomography, OCT 1 = first OCT, OCT 2 = second OCT, SD = standard deviation, EZ = ellipsoid zone, µm = micrometer, % = percent, RPE = retinal pigment epithelium, and mm3 = cubic micrometer. Central subfield is equivalent to a distance of 0.5 mm from the fovea (inner circle). Parafoveal is equivalent to a distance of 1.0 mm from the fovea (outer circle). Difference for en face percentage of attenuation is stated as an absolute change in percentage.

## Data Availability

The data are contained within this article.

## References

[1] Mavrikakis I., Sfikakis P.P., Mavrikakis E., Rougas K., Nikolaou A., Kostopoulos C., Mavrikakis M. (2003). The Incidence of Irreversible Retinal Toxicity in Patients Treated with Hydroxychloroquine A Reappraisal. Ophthalmology.

[2] Marmor M.F., Kellner U., Lai T.Y., Melles R.B., Mieler W.F. (2016). Recommendations on Screening for Chloroquine and Hydroxychloroquine Retinopathy (2016 Revision). Ophthalmology.

[3] Yusuf I.H., Sharma S., Luqmani R., Downes S.M. (2017). Hydroxychloroquine Retinopathy. Eye.

[4] Lally D.R., Heier J.S., Baumal C., Witkin A.J., Maler S., Shah C.P., Reichel E., Waheed N.K., Bussel I., Rogers A. (2016). Expanded Spectral Domain-OCT Findings in the Early Detection of Hydroxychloroquine Retinopathy and Changes Following Drug Cessation. Int. J. Retin. Vitr..

[5] Marmor M.F. (2012). Comparison of Screening Procedures in Hydroxychloroquine Toxicity. Arch. Ophthalmol..

[6] Marmor M.F., Hu J. (2014). Effect of Disease Stage on Progression of Hydroxychloroquine Retinopathy. Jama Ophthalmol..

[7] Marmor M.F., Melles R.B. (2014). Disparity between Visual Fields and Optical Coherence Tomography in Hydroxychloroquine Retinopathy. Ophthalmology.

[8] Greenstein V.C., Amaro-Quireza L., Abraham E.S., Ramachandran R., Tsang S.H., Hood D.C. (2015). A Comparison of Structural and Functional Changes in Patients Screened for Hydroxychloroquine Retinopathy. Doc. Ophthalmol..

[9] Kellner S., Weinitz S., Kellner U. (2009). Spectral Domain Optical Coherence Tomography Detects Early Stages of Chloroquine Retinopathy Similar to Multifocal Electroretinography, Fundus Autofluorescence and Near-infrared Autofluorescence. Brit. J. Ophthalmol..

[10] Browning D.J., Lee C. (2014). Relative Sensitivity and Specificity of 10-2 Visual Fields, Multifocal Electroretinography, and Spectral Domain Optical Coherence Tomography in Detecting Hydroxychloroquine and Chloroquine Retinopathy. Clin. Ophthalmol..

[11] Cukras C., Huynh N., Vitale S., Wong W.T., Ferris III F.L., Sieving P.A. (2015). Subjective and Objective Screening Tests for Hydroxychloroquine Toxicity. Ophthalmology.

[12] Ahn S.J., Joung J., Lim H.W., Lee B.R. (2017). Optical Coherence Tomography Protocols for Screening of Hydroxychloroquine Retinopathy in Asian Patients. Am. J. Ophthalmol..

[13] Rodriguez-Padilla J.A., Hedges T.R., Monson B., Srinivasan V., Wojtkowski M., Reichel E., Duker J.S., Schuman J.S., Fujimoto J.G. (2007). High-Speed Ultra–High-Resolution Optical Coherence Tomography Findings in Hydroxychloroquine Retinopathy. Arch. Ophthalmol..

[14] Chen E., Brown D.M., Benz M.S., Fish R.H., Wong T.P., Kim R.Y., Major J.C. (2010). Spectral Domain Optical Coherence Tomography as an Effective Screening Test for Hydroxychloroquine Retinopathy (the “flying saucer” sign). Clin. Ophthalmol..

[15] Pasadhika S., Fishman G.A., Choi D., Shahidi M. (2010). Selective Thinning of the Perifoveal Inner Retina as an Early Sign of Hydroxychloroquine Retinal Toxicity. Eye.

[16] Ugwuegbu O., Uchida A., Singh R.P., Beven L., Hu M., Kaiser S., Srivastava S.K., Ehlers J.P. (2019). Quantitative Assessment of Outer Retinal Layers and Ellipsoid Zone Mapping in Hydroxychloroquine Retinopathy. Brit. J. Ophthalmol..

[17] Allahdina A.M., Chen K.G., Alvarez J.A., Wong W.T., Chew E.Y., Cukras C.A. (2019). Longitudinal Changes in Eyes with Hydroxychloroquine Retinal Toxicity. Retina.

[18] Pham B.H., Marmor M.F. (2019). Sequential Changes in Hydroxychloroquine Retinopathy up to 20 Years After Stopping the Drug. Retina.

[19] Garrity S.T., Jung J.Y., Zambrowski O., Pichi F., Su D., Arya M., Waheed N.K., Duker J.S., Chetrit Y., Miserocchi E. (2019). Early Hydroxychloroquine Retinopathy: Optical Coherence Tomography Abnormalities Preceding Humphrey Visual Field Defects. Brit. J. Ophthalmol..

[20] de Sisternes L., Hu J., Rubin D.L., Marmor M.F. (2016). Analysis of Inner and Outer Retinal Thickness in Patients Using Hydroxychloroquine Prior to Development of Retinopathy. JAMA Ophthalmol..

[21] de Sisternes L., Hu J., Rubin D.L., Marmor M.F. (2015). Localization of Damage in Progressive Hydroxychloroquine Retinopathy on and off the Drug: Inner Versus Outer Retina, Parafovea Versus Peripheral FoveaRetinal Layers in Progressive HCQ Retinopathy. Investig. Ophth. Vis. Sci..

[22] Modi Y.S., Au A., Parikh V.S., Ehlers J.P., Schachat A.P., Singh R.P. (2016). Volumetric Single-Layer Inner Retinal Analysis in Patients with Hydroxychloroquine Toxicity. Retina.

[23] Arepalli S., Srivastava S.K., Hu M., Kaiser P.M., Dukles N., Reese J.L., Ehlers J.P., Lewin A.S. (2018). Assessment of Inner and Outer Retinal Layer Metrics on the Cirrus HD-OCT Platform in Normal Eyes. PLoS ONE.

[24] Itoh Y., Vasanji A., Ehlers J.P. (2016). Volumetric Ellipsoid Zone Mapping for Enhanced Visualisation of Outer Retinal Integrity with Optical Coherence Tomography. Brit. J. Ophthalmol..

[25] Banaee T., Singh R.P., Champ K., Conti F.F., Wai K., Bena J., Beven L., Ehlers J.P. (2018). Ellipsoid Zone Mapping Parameters in Retinal Venous Occlusive Disease with Associated Macular Edema. Ophthalmol. Retin..

[26] Arepalli S., Traboulsi E.I., Ehlers J.P. (2018). Ellipsoid Zone Mapping and Outer Retinal Assessment in Stargardt Disease. Retina.

[27] Lavine J.A., Srivastava S.K., Dukles N., Reese J.L., Ehlers J.P. (2020). Longitudinal Ellipsoid Zone and Subretinal Fluid Mapping Following Ocriplasmin Injection in the Prospective Observational ORBIT Trial. Brit. J. Ophthalmol..

[28] Itoh Y., Ehlers J.P. (2016). Ellipsoid Zone Mapping and Outer Retinal Characterization After Intravitreal Ocriplasmin. Retina.

[29] Ehlers J.P., Uchida A., Hu M., Figueiredo N., Kaiser P.K., Heier J.S., Brown D.M., Boyer D.S., Do D.V., Gibson A. (2019). Higher Order Assessment of OCT in Diabetic Macular Edema from the VISTA Study: Ellipsoid Zone Dynamics and the Retinal Fluid Index. Ophthalmol. Retin..

[30] Cakir A., Ozturan Ş.G., Yildiz D., Erden B., Bolukbasi S., Tascilar E.K., Yanmaz M.N., Elcioglu M.N. (2019). Evaluation of Photoreceptor Outer Segment Length in Hydroxychloroquine Users. Eye.

[31] Kalra G., Talcott K.E., Kaiser S., Ugwuegbu O., Hu M., Srivastava S.K., Ehlers J.P. (2022). Machine Learning–Based Automated Detection of Hydroxychloroquine Toxicity and Prediction of Future Toxicity Using Higher-Order OCT Biomarkers. Ophthalmol. Retin..

[32] Talcott K.E., Kalra G., Cetin H., Cakir Y., Whitney J., Budrevich J., Reese J.L., Srivastava S.K., Ehlers J.P. (2024). Automated Evaluation of Ellipsoid Zone At-Risk Burden for Detection of Hydroxychloroquine Retinopathy. J. Pers. Med..

[33] De Silva T., Jayakar G., Grisso P., Hotaling N., Chew E.Y., Cukras C.A. (2021). Deep Learning-Based Automatic Detection of Ellipsoid Zone Loss in Spectral-Domain OCT for Hydroxychloroquine Retinal Toxicity Screening. Ophthalmol. Sci..

